# Factors associated with nonadherence to diet and physical activity among nepalese type 2 diabetes patients; a cross sectional study

**DOI:** 10.1186/1756-0500-7-758

**Published:** 2014-10-24

**Authors:** Janaki Parajuli, Farzana Saleh, Narbada Thapa, Liaquat Ali

**Affiliations:** Department of Community Medicine, Nepalgunj Medical College, Teaching Hospital, Koholpur, Banke Nepal; Department of Community Nutrition, Bangladesh University of Health Sciences (BUHS), Dhaka, Bangladesh; Department of Community Medicine, Nepal Army Institute of Health Sciences, Kathmandu, Nepal; Department of Biochemistry and Cell Biology, Bangladesh University of Health Sciences (BUHS), Dhaka, Bangladesh

**Keywords:** Nonadherence, Adherence, Diet, Physical activity, Type 2 diabetes

## Abstract

**Background:**

Nonadherence to diet and physical activity is a major problem in the management of diabetes mellitus and its complications. This study was undertaken to measure the factors associated with nonadherence to diet and physical activity advice among Nepalese type 2 diabetic patients.

**Methods:**

An analytical cross-sectional study was conducted among type 2 diabetic patients (age, M ± SD, 54.4 ± 11.5 yrs) and interviewed using three days recall method for dietary history and Compendium of Physical Activity for physical activity. Data were analysed by univariate and multivariate statistics.

**Results:**

Out of 385 patients, 87.5% were nonadherent and 12.5% poorly adherent to dietary advice. 42.1% were nonadherent, 36.6% partially adherent while 21.3% good adherent to physical activity. Adherence to dietary advice was higher in males than females (M ± SD, 33 ± 16.7 vs 27 ± 15.5, p = 0.001), those staying nearer to hospital than farther (M ± SD, 32 ± 18.6 vs 28 ± 13.5, p = 0.013), those advice by physician than others (p = 0.001) and from nuclear family than joint and extended (p = 0.001). With increasing age, dietary advice adherence decreased (p = 0.06) and was positively correlated with the knowledge about diabetes mellitus (r = 0.115, p = 0.024). Physical activity adherence was higher in those with positive family history of diabetes than others (M ± SD, 74 ± 24.2 vs 65 ± 23.6, p = 0.001), upper middle socioeconomic class respondents than lower ones (p = 0.047) and from extended family than nuclear or joint ones (p = 0.041). Divorced were more nonadherent to physical activity than married and widowed patients (p = 0.021).

**Conclusions:**

Determinants of nonadherence to dietary advice: Female gender, increasing age, joint or extended family members, farther distance from hospital, poor knowledge about diabetes mellitus and advice by others than physicians. Determinants for nonadherence to physical activity: negative family history of DM, divorced status, lower socioeconomic class.

## Background

Diabetes – A global epidemics and a serious public health problem. 382 million people have diabetes in 2013; by 2035 this will rise to 592 million. The number of people with type 2 diabetes is increasing in every country. 80% of people with diabetes live in low- and middle-income countries. The greatest number of people with diabetes are between 40 and 59 years of age [1]. In Nepal the number of diabetic patients was 436,000 in 2000 and it was projected be 1,328,000 in Nepal by 2030 [2]. Healthy dietary habits and lifestyle modifications- the cornerstones of type 2 diabetes prevention and management [3]. The Diabetic Prevention Program suggested that dietary and physical activity changes to produce a 5-7% weight loss successfully maintains glycemic control in people diagnosed with type 2 diabetes [4]. Adherence to lifestyle modification recommendations lessens the disease burden and reduces the morbidity and mortality associated with type 2 diabetic complications.

One study in Egypt showed that only 2.2% of the respondents adhered with dietary regimen while no one reported regular compliance with exercise regimen [5]. In another study done in US, it, was found that 52% diabetic subjects followed the dietary advice [6]. A study done in Alexandria showed that only 10.7% had good compliance level, 18% have poor compliance and majority 78.3% are noncompliant to overall diet and physical activity [7]. Similar results were also found in studies conducted in South East Asia. In a Bangkok based study, the proportion of cases with good adherence to physical exercise and diet regimen were 31.7% and 54.3% [8]. In a study conducted in India, dietary prescriptions and exercises were followed regularly by only 37% and 35% of patients [9]. Rapid socioeconomic development, urbanization, globalization, and an expanding number of fast food outlets, leading to unusual consumption and over dependence, may be contributing to factors influencing adherence to lifestyle modification recommendations amongst type 2 diabetes mellitus patients [3]. The extent of nonadherence to diet and physical activity and the factors influencing it are different in different populations in Nepal. This may be due to difference in lifestyle, culture, eating habits, knowledge and beliefs. Moreover dietary adjustment and lifestyle modification are the integral part of management of diabetes. Since management of the disorder creates a great physical, psychological and socioeconomic burden on the family and the society, priority should be given on the preventive aspects of disorders with diet and lifestyle modifications. This study aims to assess the proportion of nonadherence to diet and physical activity among type 2 diabetic patients and the factors associated with nonadherence to diet and physical activity advices.

## Methods

**Figure 1:**
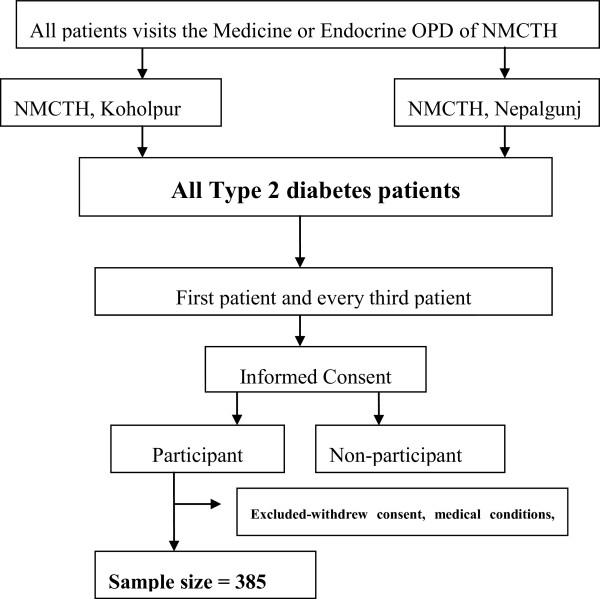
**Flow chart showing the sampling technique.**

An analytical study with cross-sectional design was adopted and 385 type 2 diabetic patients, diagnosed for at least 3 months, were selected from tertiary level care hospital using the systematic random sampling method (Figure 1) The minimum required sample size was calculated as 358 using formula n = z2pq/d2 (where, n = the required sample size; p = the prevalence of nonadherence to diet i.e. 63% and physical activity is 65% [9], i.e. q = 1-p and d = error (precision) i.e. 5%. 385 was taken as the sample size of the study. Data were collected by a pre-tested, interviewer administered questionnaire. Information on sociodemographic characteristics, health care delivery system and clinical characteristics Knowledge about diabetes was poor if <40% fo total score, and good if >60% of total score. Socioeconomic status was assessed using a modified version of Kuppuswamy’s scale fro used in Nepal [10]. Dietary history was taken by three days recall method and physical activity was assessed by using Compendium of Physical Activity and GPAQ scoring. Anthropometric measurements were done by using the appropriate tools.

Adherence was measured by compliance to the advice given to individual subjects. Respondents calorie intake and the macronutrient (carbohydrate, protein, fats and fiber) intake along with frequency and timing of meal were calculated. If the intake of each item was within the prescribed range of dietary intake (Calorie: 1800 Kcal ± 10%, Carbohydrate: 288 gm ± 10%, Protein: 73 gm ± 10%, Fat: 52 gm ± 10%, Fiber: 25gm ± 10%) than score was given 1, otherwise 0. All the food item scores were added and finally dietary adherence score was made. Those with > = 75% of the total score, they were good adherent, those with 50–75% of total score were poor adherent, while those with <50% were nonadherent. For physical activity, total METs value of each individual was calculated per week and was converted into METs min/week. Adherence level was scored on the basis of GPAQ scoring as >1500 METs min/week, good adherent; 600–1500 METs min/week, partial adherent and <600 METs min/week, poor adherent. Data were analysed by univariate as well as multivariate statistics. Independent *t*-test, One way ANOVA and multiple regression analysis was done to find the relationships between variables. Informed written consent was obtained from all respondents after a full explanation of the nature, purpose and procedures used for the study. This study was approved by the Institutional Review Committee of BUHS and then Ethical Review Board of BADAS and NHRC and Institutional Review Committee of NGMCTH.

## Results

**Figure 2:**
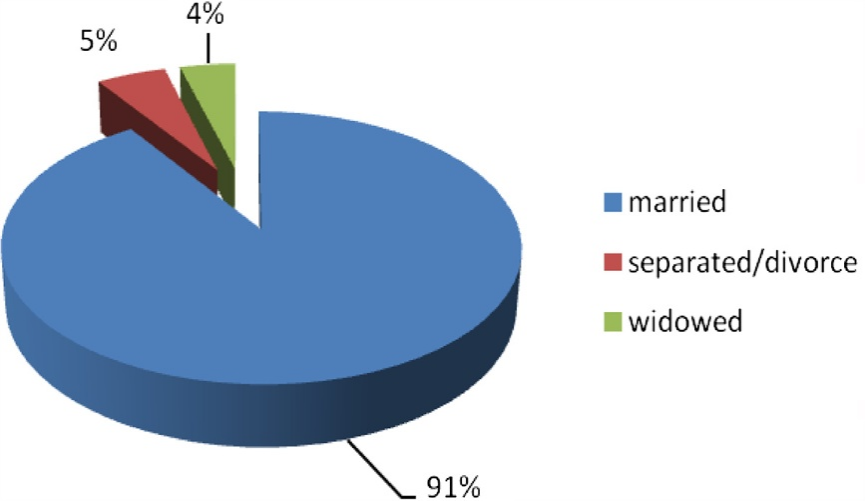
**Distribution of the respondents according to the marital status.**

**Figure 3:**
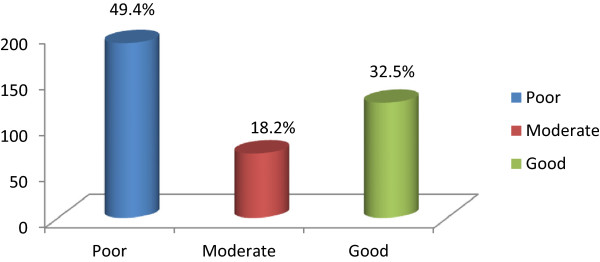
**Distribution of the respondents according to the knowledge level about type 2 diabetes.**

**Figure 4:**
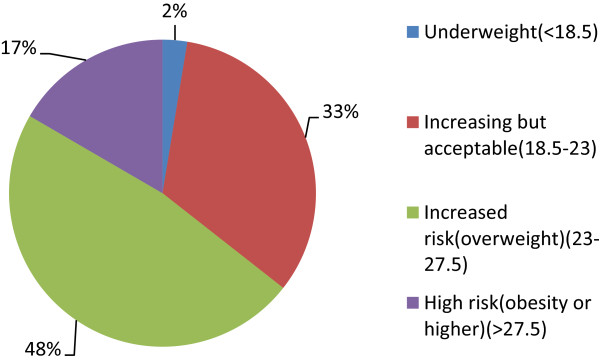
**Distribution of the respondents according to the BMI.**

A total number of 385 type 2 diabetes patients were enrolled as the study population with mean (SD) age 54.4 (11.5) years and female–male proportion of 51.4% and 48.6% respectively where 29% were aged between 41–50 years age group. 35.1%of the respondents were illiterate and 48% were employed. Only 39% had income greater than 10,000 rupees per month. Almost 50% of the respondents belonged to nuclear family. (Table 1) 91% of respondents were married (Figure 2). 49.9% of the respondents had poor knowledge level, 18.2% had moderate while 32.5% had good knowledge about type 2 diabetes mellitus (Figure 3). Majority of the respondents 184 (48%) were obese, 64 (16%) were overweight and 127 (33%) were in the acceptable (normal) BMI [11] (Figure 4). The mean BMI was 24.26 ± 3.33 (M ± SD).

**Table 1:** **Distribution of the respondents according to the sociodemographic characteristics (n = 385)**

Variables	Number (%)
**Age**	
<40	51 (13.2)
41-50	114 (29.6)
51-60	107 (27.8)
61-70	81 (21.0)
>71	32 (8.3)
**Mean ± SD**	54.4 ± 11.5
**Sex**	
Female	198 (51.4)
Male	187 (48.6)
**Education level**	
Illiterate	135 (35.1)
Informal education	44 (11.4)
Primary	62 (16.1)
Secondary	56 (14.5)
Higher secondary and above	88 (22.9)
**Occupation**	
Housewife	151 (39.2)
Employed	188 (48.8)
Unemployed	46 (11.9)
**Income per month (Rs)**	
<5000	160 (27.5)
5000-10000	129 (33.5)
>10000	150 (39.0)
**Economic status**	
Middle upper (II)	248 (64.4)
Lower middle (III)	137 (35.6)
**Types of family**	
Nuclear family	194 (50.4)
Joint	155 (40.3)
Extended	36 (9.4)
**Smoking habit**	
Currently yes	89 (23.1)
Nonsmokers	296 (76.9)
**Alcohol habits**	
Currently yes	64 (16.6)
Nondrinkers	321 (83.4)

Results are expressed by M ± SD or number (%).

**Figure 5:**
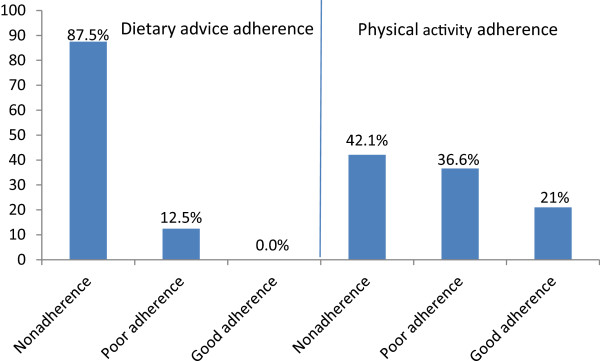
**Proportion of adherence to diet and physical activity.**

**Figure 6:**
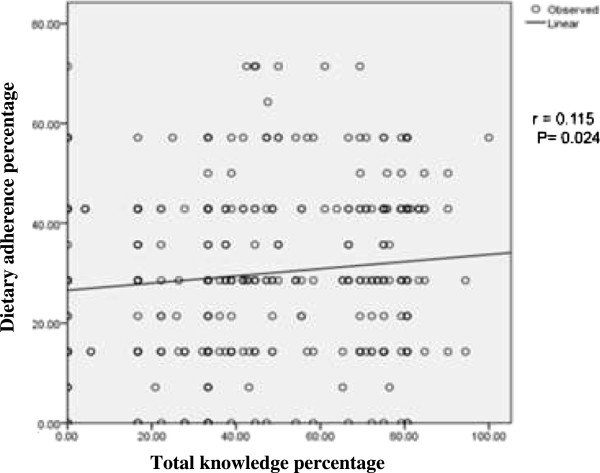
**Correlation of adherence to dietary advice with total knowledge score percentage.**

Poor adherence was seen in 12.5% of the patients while maximum 87.5% were nonadherent to dietary advice. Mean adherence of physical activity is 30 ± 16.3 (M ± SD). In physical activity majority (162)42.1% were nonadherent to physical activity advice while 36.6% had poor adherence level and proportion of adherence was good only on 21.3% of the respondents. Mean adherence of physical activity was (M ± SD, 67 ± 23.9). (Figure 5 or Table 2). Adherence level was higher in males than females (M ± SD, 33 ± 16.7 vs 27 ± 15.5, p = 0.001). With increasing age, level of dietary advice adherence decreased (p = 0.06). Respondents from nuclear family were more adherent to dietary advice than joint and extended (p = 0.001) ones. Adherence level was higher among those staying nearer to hospital than far from hospital (M ± SD, 32 ± 18.6 vs 28 ± 13.5, p = 0.013). Adherence level of dietary advice was higher among those advised by physicians than others (p = 0.001). (Table 3) Adherence level was positively correlated with the knowledge about diabetes mellitus score (r = 0.115, p = 0.024) (Figure 6).

**Table 2:** **Distribution of respondents according to the nonadherence to dietary advice and physical activity (n = 385)**

Variables	Frequency	Percent	Mean ± SD
**Dietary advice**			
Good adherence	0	0	
Poor/partial adherence	48	12.5	30 ± 16.3
Nonadherence	337	87.5	
**Physical activity**			
Good adherence	82	21.3	
Poor/partial adherence	141	36.6	67 ± 23.9
Nonadherence	162	42.1	

Results are expressed by M ± SD or number (%).

**Table 3:** **Association of adherence to dietary advice and physical activity with different variables (n = 385)**

Variables	Adherence% dietary advice (M ± SD)	Adherence% physical activity (M ± SD)
**Sex**		
Female	27 ± 15.5	66 ± 23.5
Male	33 ± 16.7	69 ± 24.4
**t/p**	**3.55/0.001** *	NS
**Advice given by**		
Physician	33 ± 16.7	68 ± 23.0
Others	24 ± 13.8	64 ± 25.8
**t/p**	**5.204/0.001** *	NS
**Advice during follow-up**		
Yes	33 ± 16.7	68 ± 23.0
No	24 ± 13.8	64 ± 25.8
t/p	**4.381/0.001** *	NS
**Age**		
<40	30 ± 14.8	71 ± 22.0
41-50	28 ± 17.3	68 ± 21.9
51-60	34 ± 15.6	69 ± 27.6
>61	28 ± 16.2	62 ± 22.7
F/p	**2.857/0.035**	**1.971/0.048**
**Marital status**		
Married (M)	29 ± 16.1	68 ± 23.9
Separate/divorce (S)	27 ± 18.1	52 ± 26.8
Widowed (W)	42 ± 15.0	69 ± 14.4
F/p	**4.643/0.010**	**3.896/0.02**
**Occupation**		
Housewife (H)	28 ± 16.3	65 ± 23.1
Employed (E)	32 ± 16.5	69 ± 24.5
Unemployed (U)	28 ± 14.8	64 ± 24.5
F/p	**2.866/0.058**	NS
**Income per mth (Rs)**		
<5000 (<5 T)	33 ± 17.0	69 ± 25.8
5000-1000 (5–10)T	28 ± 14.4	65 ± 24.7
>10000 (>10 T)	30 ± 16.3	68 ± 21.9
F/p	**2.665/0.041**	NS
**Types of family**		
Nuclear	34 ± 17.4	67 ± 21.2
Joint	26 ± 14.1	69 ± 24.9
Extended	24 ± 12.8	56 ± 31.3
F/p	**15.189/0.001**	**4.342/0.041**
**Area of residence**		
Rural (R)	27 ± 17	73 ± 25.7
Semiurban (S)	31 ± 15.7	64 ± 24.6
Urban (U)	30 ± 16.8	68 ± 19.4
F/p	NS	**5.577/0.004**
**Duration of diabetes (years)**		
<2	33 ± 17.6	66 ± 25.1
2-6	29 ± 14.6	66 ± 23.3
>6	25 ± 15.3	64 ± 23.0
F/p	**7.746/0.01**	NS
**Knowledge level**		
Poor (P)	25 ± 13.8	69 ± 22.8
Moderate (M)	32 ± 16.3	64 ± 25.3
Good (G)	36 ± 19.3	66 ± 24.6
F/p	**16.67/0.001**	NS

*Results expressed in mean ± SD. Independent *t*-test was performed as test is significance, p < 0.05 was taken as level of significance.

Results are expressed by mean ± SD. One way ANOVA (Turkey) was performed as the test of significance, p < 0.05 was taken as the level of significance.

Physical activity adherence level was higher in the respondents with positive family history of diabetes compared to those with no family history (M ± SD, 74 ± 24.2 vs 65 ± 23.6, p = 0.001). Divorced were more nonadherent to physical activity than married and widowed patients (p = 0.021). Respondents from rural area had higher level of adherence than urban and semiurban ones (p = 0.004). Upper middle socioeconomic class respondents had higher adherent level to physical activity than lower class ones (p = 0.047). Respondents belonging to extended family had higher level of nonadherence than those from nuclear or joint family (p = 0.041) (Table 3).

## Discussion

Since management of the disorder of diabetes mellitus creates a great physical, psychological and socioeconomic burden on the family and the society, priority should be given on the preventive aspects of disorders with diet and lifestyle modifications. There is no published data yet regarding factors affecting diet and physical activity nonadherence in Nepal in order to compare the results. However, results for dietary advice and physical activity compliance assessment have been found by different researchers in different countries. Nonadherence to dietary advice was higher in the current study than those in Mexican Americans (25.2%) [12], Ohio (33.4%) [13], Iran (37%) [14], Oregan (50%) [15], Calgary (55%) [16], Kuwait (63.5%) [17], Saudi Arabia (67.9%) [18], Texas (67.9%) [19], Alexandria (68%) [6] and Hungary (78.3%) [20] and lower than study done in Egypt6 which was found to be 94.3%. Comparing the current finding with the South East Asian data, nonadherence to dietary advice was seen on 45.7% and 63% on Bangkok and Indian population respectively [9, 21]. Nonadherence to physical activity is higher in the present study than those in Hungary (33.8%) [20], Ohio (32.7%) [13] and WHO study (31.7) [22] and lower than findings of UAE (53%) [23], Mexican Americans (69%) [12], Kuwait (69%) [17], Oregon (70%) [24] and Dutch (90%) [25]. Comparing the current findings with the South East Asian data, nonadherence to physical activity was seen on 68.3% and 65% on Bangkok and Indian population respectively [9, 21].

### Dietary advice nonadherence

In relation to gender, nonadherence to dietary advice of female is higher than male respectively which is statistically significant (p = 0.001). In contradiction to the present study, study done in Nigeria showed male diabetic patients seemed to have greater tendencies to forget dietary regimen than their female counterparts [26]. However the result is different from the study done in Egypt, which showed that there was minimal gender difference with no statistical differences in adherence to different aspects of the diabetic regimen [6]. The present study shows significant age difference in relation to the adherence to dietary advice.

With increasing age, the degree of compliance decreases for several reasons, most of the elderly have memory problems and decreased cognitive function. Similar result was reported by another study where adherence level decreases with increasing age [6]. Significant difference in adherence level in accordance to the duration of diabetes is seen in the present study. With increasing duration of disease degree of adherence was decreasing. This can be explained by the reason that with increase in duration of disease, patients might be fed up with the treatment and dietary regimen to follow. This observation is consistent with the other studies which showed comparing acute and chronic forms of diseases in which chronicity was associated with poor compliance, increasing duration was found to be predictive of decreasing total compliance score [27]. Patients advised by physicians are more adherent to dietary advice than others. The reason might be that patients believe physicians more than the nurses and the dietitians. However, in the study done in India it was seen that those who visited the dieticians were better able to adhere to the diet than those who had merely been advised by the physicians [28]. It can be explained by the fact that dieticians necessarily have broader knowledge with the advice on healthy food options, cooking methods, practical guidance to deal with lifestyle issues.

In the present study the respondents from the nuclear family has higher adherence level than joint or extended family and the difference is statistically significant. Less family members may be economically secure about various food options which they require. Similar result was shown in the study where 69% of the patients who belong to nuclear family were following dietary advice for full duration of diabetes [28]. Difference in the adherence level is found on the basis of economic status. Higher adherence level is seen on respondents belonging to upper middle class than lower.

This study shows a statistically significant difference between marital status and nonadherence level. Widowed are more adherent to dietary advice than married and separated. The reason might be widowed are more free of any responsibilities and concerned about their health than others.

Adherence to dietary advice is higher in those respondents who are nearer to hospital than who are far and the difference was statistically significant (p = 0.013). The reason might be, patients who were nearer have frequent visits to the health care provider, with better follow-up than those who are far. Similar reason was shown in the study by Terri Travis that patients who visited the dietician more often and had more follow-ups were better able to adhere to the diet than those who visited the dieticians less often and didn’t have follow up sessions [29]. In this study, almost half (49.2%) has poor knowledge about type 2 diabetes. There is a statistically significant positive correlation between knowledge about type 2 diabetes and adherence to dietary advice. If the knowledge is poor, nonadherence to dietary advice is higher while adherence increases with increased knowledge level. A study done in Egypt also showed a finding similar with the current study that the level of compliance increased with the improvement of the patient’s level of knowledge about diabetes [6, 30]. On the other hand, findings of a study conducted in China indicated that there was no association between the knowledge of diabetes and compliance [31]. Formal diabetes education participation was done by only 23% of the patients. They showed to have higher adherence to dietary advice than those who didn’t participated. Planning a realistic diet and exercise program was favored as a solution for the lack of motivation and will power which further improve the adherence level [32]. One alarming proportion is found in the another study population: 62.0% of diagnosed diabetes didn’t have access to health education programmes and they have high nonadherence level [33].

### Physical activity nonadherence

Regarding family history, only 84 (21.4%) of the respondents have family history of diabetes. Level of nonadherence to physical activity differs with family history of diabetes which is statistically significant. Higher level of adherence is seen in those who have family history of type 2 diabetes than those who do not. Our study results contradict with the study done in University of Glasglow [34].

The present study shows statistical significant differences in adherence level to physical activity in relation to marital status. Nonadherence level is lower in married than divorce or separated. This might be due to the reason that married respondents get better spouse and family members support than divorce or separated. One study showed that the patients who were not supported by the spouse and the family members, only 14.2% were adherent to the exercise regimen [11]. However, one study showed no any consistent relationship of physical activity adherence with the marital status [35].

Area of resistance also shows statistical differences in the adherence level of physical. Similar result was shown in the study where higher nonadherence in physical activity was seen in people living in ubran [8]. The present result shows contradiction with the result shown on study of US women i.e. women living in rural regions were more likely to be completely inactive during leisure time than were women living in urban areas [36]. Physical activity nonadherence differs significantly with the types of family. The respondents from extended family have higher level of nonadherence than those from nuclear and joint family. This might be due to the reason that family members couldn’t give support for the lifestyle changes. However, social support is useful and helps patients learn greater self acceptance, develop new norms for interpersonal relationship and manage the schedule for physical activity [34]. Physical activity nonadherence varies with the socioeconomic status which is statistically significant. Lower middle class has higher level of nonadherence to physical activity than upper middle class respondents. This result is consistent with the study done in Egypt where poor income group people often had higher nonadherence level to physical activity than others [8]. Lack of available facilities and cost are additional barriers to low income women participating in physical activity [37].

## Conclusions and recommendations

The vast majority (87.5%) of type 2 diabetic patients in Nepalgunj area of Nepal are nonadherent to dietary advice and even the remaining ones are only poorly adherent. Adherence to physical activity in the same population is much better (with corresponding nonadherence of 42.1%), but still only one-fifth (21.3%) of the population have good adherence level and the remaining (36.6%) are only poorly adherent.

All stakeholders including clinicians, dietitians, health educationists and policy makers should be made aware about the alarmingly high proportion of nonadherence to dietary and physical activity advices among diabetic population of Nepalgunj area. Dietary and physical activity advices for diabetes should be tailored to individual patients with particular focus on gender, marital status, family size, socioeconomic status, knowledge about diabetes, family history of diabetes, urban–rural origin and distance from the hospital. Large scale studies, particularly with prospective design, should be undertaken to have more in-depth knowledge on the level and determinants of nonadherence to diet and physical activity advices in individual Nepalese type 2 diabetic populations.

### Strength and limitations of the study

The result of this study should however be considered in line with some limitations which include; self reported dietary history and physical activity may be subjective and might underestimate patients nonadherence status. Only patients from the tertiary health care facility were studied limiting the geographical diversity and implying difficulty in generalizing finding to the Nepalese diabetes population. Systemic sampling technique was used for patient selection to minimize the bias. Nonetheless, the study findings provide valuable information suggesting the need of routinely observing the reasons for diet and lifestyle nonadherence among type 2 diabetes patients. This study will make an important contribution to the prescription of diet and physical activity measures in the management of type 2 diabetes. Informing health care providers about these study findings would increase their commitment to the inclusion of therapeutic lifestyle measures as part of the intervention to be used in managing people with type 2 diabetes mellitus.

## Abbreviations

ANOVA: Analysis of variance
BADAS: Diabetes Association of Bangladesh
BMI: Body metabolic index
BUHS: Bangladesh University of Health Sciences
DM: Diabetes mellitus
FFQ: Food frequency questionnaire
GPAQ: Global physical activity questionnaires
METs: Metabolic equivalents
NHANES: National Health and Nutrition Examination Survey
NHRC: Nepal Health Research Council
NGMCTH: Nepalgunj Medical College Teaching Hospital
OHA: Oral hypoglycemic agent
OGTT: Oral glucose tolerance test
OPD: Out patient department
SD: Standard deviation
SEA: South East Asia
SPSS: Statistical package for social sciences
TG: Triglyceride
USA: United States of America
WHO: World Health Organization.
